# EBV-microRNAs as Potential Biomarkers in EBV-related Fever: A Narrative Review

**DOI:** 10.2174/1566524023666221118122005

**Published:** 2023-12-01

**Authors:** Wei-ting Wang, Yun Yang, Yang Zhang, Yi-ning Le, Yu-lin Wu, Yi-yi Liu, Yan-jie Tu

**Affiliations:** 1 School of Acupuncture-moxibustion and Tuina, Shanghai University of Traditional Chinese Medicine, Shanghai (201203), China;; 2 Information Center of Science and Technology, Shanghai Innovation Center of TCM Health Service, Shanghai University of Traditional Chinese Medicine, Shanghai (201203), China;; 3 National Key Laboratory of Medical Immunology & Institute of Immunology, Second Military Medical University, Shanghai (200433), China;; 4 Longhua Hospital, Shanghai University of Traditional Chinese Medicine, Shanghai (200032), China;; 5 Department of Febrile Disease, Basic Medical College, Shanghai University of Traditional Chinese Medicine, Shanghai (201203), China

**Keywords:** Epstein-Barr, virus, microRNAs, therapeutic targets, EBV-related fevers, biomarker

## Abstract

At present, timely and accurate diagnosis and effective treatment of Epstein-Barr Virus (EBV) infection-associated fever remain a difficult challenge. EBV encodes 44 mature microRNAs (miRNAs) that inhibit viral lysis, adjust inflammatory response, regulate cellular apoptosis, promote tumor genesis and metastasis, and regulate tumor cell metabolism. Herein, we have collected the specific expression data of EBV-miRNAs in EBV-related fevers, including infectious mononucleosis (IM), EBV-associated hemophagocytic lymphohistiocytosis (EBV-HLH), chronic active EBV infection (CAEBV), and EBV-related tumors, and proposed the potential value of EBV-miRNAs as biomarkers to assist in the identification, diagnosis, and prognosis of EBV-related fever, as well as therapeutic targets for drug development.

## 1 INTRODUCTION

As a successful member of the most familiar human DNA viruses, Epstein-Barr Virus (EBV) plays a part in the induction of various diseases, which include infectious mononucleosis (IM), Epstein-Barr Virus-associated hemophagocytic lymphohistiocytosis (EBV-HLH), and chronic active Epstein-Barr Virus infection (CAEBV), with over 90% of the global adult population infected [1]. Primary infection of the virus, which is mainly transmitted by salivary aerosol exposure [2], generally occurs in the oral cavity, chiefly infecting B lymphocytes and relatively few epithelial cells [3]. Following acute infection, the lifelong persistence of EBV in hosts is achieved *via* the strategy of latency [4]. In immunocompetent individuals, EBV reactivation is inhibited by effective cytotoxic cellular immunity [5], while in immunocompromised patients, the expression of BZLF1 and BRLF1, two critical immediate-early (IE) genes of EBV-producing transactivator proteins that activate the cis-acting element oriLyt initiating viral lytic replication, triggers the switch from the latent to the lytic phase [6-8].

EBV was the first virus identified to encode viral microRNAs [9, 10]. MicroRNAs (miRNAs), a group of small non-coding RNAs, exert suppressive effects on target mRNAs and play a vital role in gene expression through posttranscriptional regulation [11-14]. Discovering effective diagnostic and therapeutic targets is critical to precise treatment and better outcome. MicroRNAs may act as measurable epigenomic biomarkers [15], which indicate biological or pathogenic processes or the body’s responses to an exposure or intervention (*e.g*., therapeutic treatments) [16, 17]. In this review, the respective implications of the differential expression of EBV-encoded miRNAs in EBV-associated fever will be considered in general, along with their potential significance, not only as sensitive indicators for the biological detection but also as therapeutic targets.

## 2 GENETIC CHARACTERISTICS OF EBV-miRNAs

**Fig. (1):**
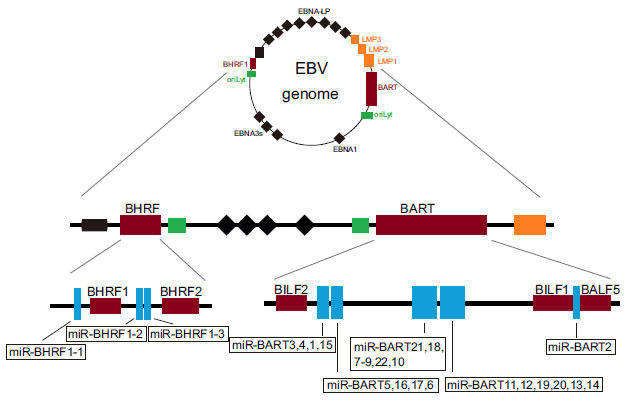
Schematic diagram of the genomic position of EBV-miRNAs. EBV can encode 25 precursor miRNAs (pre-miRNAs) located within 2 clusters of the genome: BART cluster and BHRF1 cluster. The location of 44 mature miRNAs processed by 25 pre-miRNAs is shown in the figure.

**Fig. (2):**
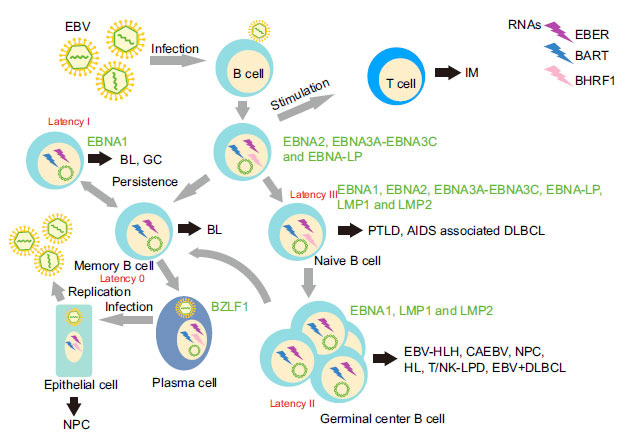
Models of Epstein–Barr virus infection and related diseases. The transient proliferation of EBV-positive B cells and over-reaction of cytotoxic T cells (CD4 + and CD8 + T lymphocytes) cause infectious mononucleosis [102]. According to the germinal center model [103], EBV infection causes growth transformation (latency III) of naive B cells in local lymphoid tissues, during which all EBV nuclear antigens (EBNAs), EBV encoded small RNAs (EBERs) and latent membrane proteins (LMPs) are expressed, thus inducing the T-cell-mediated immune reaction. At this moment, a portion of the latency III cells is transformed into germinal centre B cells that express LMP-1, LMP-2 and EBNA-1 (latency II) [2, 104]. They eventually become memory B cells that carry EBV viral genes, which express no viral proteins [2, 104] (latency 0) that enables their escape from host immune recognition [103]. EBV-latently infected memory B cells enter the peripheral blood circulation to form a lifelong persistent and asymptomatic infection [105]. During their stable proliferation *in vivo*, memory B cells undergo cell division (latency I) [2] and express the EBNA-1 gene to facilitate viral DNA replication. Different stages of latent infection can be associated with various lymphoproliferative diseases (LPD) and lymphoma [106]. When memory B cells at the pharyngeal lymphatic ring differentiate into plasma cells, they will reactivate EBV lytic infection through the expression of BZLF1, the key immediate-early (IE) gene of EBV [7, 8], producing new infectious virus particles. Nasopharyngeal carcinoma is associated with infected epithelial cells. IM, infectious mononucleosis; PTLD, post-transplant lymphoproliferative disorder; AIDS, acquired immunodeficiency syndrome; DLBCL, diffuse large B-cell lymphoma; EBV-HLH, Epstein-Barr virus-associated hemophagocytic lymphohistiocytosis; CAEBV, chronic active Epstein-Barr virus infection; NPC, nasopharyngeal carcinoma; HL, Hodgkin's lymphoma; T/NK LPD, T/NK-cell lymphoproliferative disease; BL, Burkitt's lymphoma; GC, gastric carcinoma; EBER, EBV encoded small RNA; BHRF1, Bam HI fragment H rightward open reading frame 1; BART, Bam HI fragment A rightward transcript; EBNA, EBV nuclear antigen; EBNA-LP, EBNA leader peptide; LMP, latent membrane protein.

Based on the miRNA database (http://www.mirbase.org/), Pfeffer *et al.* reported that EBV could encode 25 precursor miRNAs (pre-miRNAs), which generate 44 mature miRNAs after cleavage [9]. While the smaller Bam HI fragment H rightward open reading frame 1 (BHRF1)-cluster encodes 3 pre-miRNAs, which are processed into 4 mature miRNAs, the larger Bam HI fragment A rightward transcript (BART)-cluster encodes 22 pre-miRNAs, which are processed into 40 mature miRNAs (Fig. **1**) [18, 19]. EBV switches between latent and lytic infection cycles (Fig. **2**). The latent infection can be divided into at least four distinct stages (latency 0, latency I, latency II, and latency III). BHRF1 miRNAs show high abundance in type III latency and lytically-infected cells but are particularly undetectable in type I and II latency [20-22]. On the contrary, BARTs are found in all EBV-positive cell lines [23]. EBV-miRNAs employ three means of migrating into the host circulatory system: the passive release of broken cells, the transcellular transport of exosomes, and the combination with RNA binding protein. After invading adjacent endothelial cells, viral miRNAs guide gene silencing by blocking mRNA translation and/or activating mRNA degradation [18, 19], and thus are closely related to tumorigenesis, immune escape, inflammation, latent infection, and viral lytic replication [24-26]. The stable and sustained EBV-miRNAs expression in the peripheral blood and infected cells, as well as the diverse functions EBV-miRNAs exert, indicate its latent capacity for disease diagnosis [27, 28]. Clinical data have shown that common EBV detection methods and treatments have their limitations in treating EBV-related fever [29-33].

## 3 THE INFLUENCE OF EBV-miRNAs ON IMMUNE HOMEOSTASIS

### 3.1 EBV-miRNAs Regulate Inflammation

EBV-miRNAs are crucial immunomodulatory factors that target multiple inflammation-related pathways, resulting in the hyper-activation and inhibition of inflammatory reactions [25, 34]. On the one hand, EBV-miRNAs can initiate the inflammatory cascade and evoke an uncontrollable inflammatory process called “cytokine storm”. The resultant elevated production of various pro-inflammatory cytokines (*i.e*., TNF-α, IFN-γ, IL-1, and IL-6) triggers the massive action and monoclonal proliferation of EBV-infected cytotoxic T lymphocytes (CTL) and macrophages [35, 36]. For example, high amounts of BART3-3p in EBV-HLH [37] were deduced to upregulate the IL-6 level *via* targeting importin 7 (IPO7) [38], an important receptor for the AP-1 member c-Jun [39, 40].

On the other hand, present documents have suggested the essential role of EBV-miRNAs in restricting inflammation responses. The exosome-mediated transcellular transmission (from EBV-positive B cells to EBV non-infected B cells) of BART15-3p resulted in the reduction of IL-18 and IL-1β in inflammasomes *via* targeting the miR-223 binding site in the 3'-untranslated region (UTR) of NLRP3 [41]. The significant down-regulation of IL-6 receptor genes (IL-6 signal transducer and IL-6 receptor α) is demonstrated to be related to BART-6-3p in Burkitt lymphoma [42]. BART16 blocked the TNFα-mediated activation of the NFκB signaling pathway by silencing TRIM8 (Tripartite Motif Containing 8) [38]. Skinner *et al*. found that IL-1 receptor-1, which conjugates with IL-1β and induces pro-inflammatory actions, was downregulated by cellar BHRF1-2-5p during EBV infection [43]. Data mining on the basis of AGO PAR-CLIP experiments speculated that various virus-encoded miRNAs might affect interferon signaling. The prediction results suggested BART1, 3, 5, 10, 13, 14 and 19 as interference in the production of IFN-α mediated by type I IFN–driven pathway, whereas BART1, 2, 3, 7, 16, 17 and 22 negatively regulated the downstream effect of type I IFN signaling. Two vital factors of the type-I IFN pathway, FBOX21 and TRIM65, were considered as predicted targets of BART21-5p and 7-3p, respectively [38]. BART16 dampens CREB-mediated IFN signaling *via* direct downregulation of CREB-binding protein in EBV-transformed B cells and gastric cancer cells [44]. The amplification of two LMP1-mediated inhibitory immune checkpoint ligands, PD-L1 and PD-L2, was fine-tuned by miRNA-BHRF1-2-5p to achieve context-dependent immunomodulatory effects [45]. The over-expression of BART6-3p dampened the innate immune responses through RIG-I signaling, and thus, specifically downregulated IFN-β production [46].

### 3.2 EBV-miRNAs Block Antigen Presentation

Besides modulating innate immunity by controlling inflammation, EBV-encoded miRNAs play a promotive part in immune evasion by interfering with antigen processing and presentation. Interference with major histocompatibility complex (MHC) antigen presentation enables viral miRNAs to evade the adaptive immune response [47]. In nasal NK-cell lymphoma, BART8 and 20-5p suppressed the IFN-γ/STAT1 signaling pathway, resulting in blockage of MHC class-I antigen presentation to CD8^+^ T cells [48]. MHC Class I Polypeptide-Related Sequence B (MICB) in EBV-immortalized lymphoblastoid cell lines (LCL) was speculated to be the potential target of BART1-3p and 3 [49]. BHRF1-2, BART1 and 2 regulated the secretion of lysosomal enzymes, including cathepsin B (CTSB), legumain (LGMN) and IFN-γ-inducible lysosomal thiol reductase (IFI30), to interfere with MHC class-II antigen presentation [50]. BART1, 2, 22 and BHRF1-2 were specific in the suppression of IL-12 secretion and the differentiation of CD4^+^ into Th1-type cells, thus interfering with MHC class-II antigen processing and presentation [51].

### 3.3 EBV-miRNAs Contribute to Interference with Immune Surveillance of T Cells

**Fig. (3):**
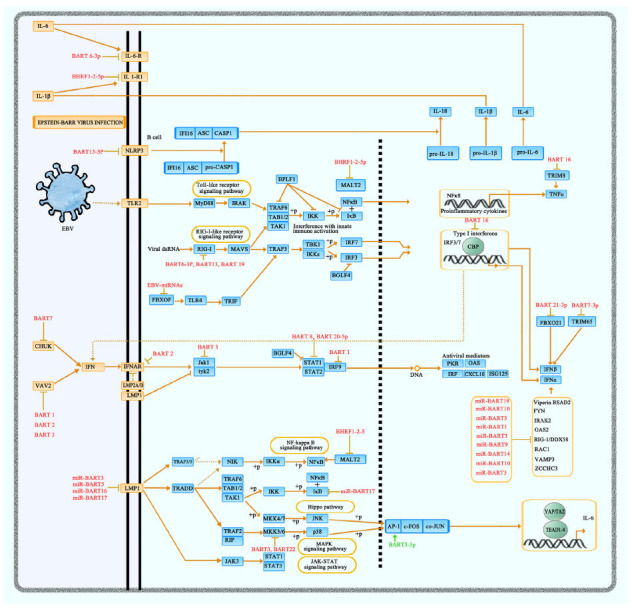
EBV-miRNAs modulating inflammatory signaling pathways. This figure illustrates the strategies EBV-encoded miRNAs apply to regulate the inflammatory signaling pathways in B cells during EBV infection. **1**) NF-κB pathway: BART15-3p blocks the maturation of IL-1β, IL-16, and IL-18 by inhibiting NLRP3 recognition in NLRP3- NF-κB pathway. BHRF1-2-5p and BART17 serve as inhibitors of protein kinases in the NF-κB pathway. **2**) Type I interferons signaling pathway: BART6-3p, 13 and 19 block RIG-I recognition of viral double-stranded (ds) RNA, which subsequently perturbs the activation of type I interferons signaling pathway. BART1, 2, 3, 7, 8, and 20-5p negatively regulate the downstream effect of type I IFN signaling. **3**) MAPK pathway: BART3 and 22 hamper MKK3/6 phosphorylation in p38. **4**) JAK-STAT pathway: BART8 and 20-5p suppress STAT1 activity. **5**) Hippo pathway: BART3-3p triggers the activation of AP-1 trimer, thereby upregulating IL-6 expression. BART16 directly targets CREB binding protein (CBP) to block CREB-dependent transcription. BART1, 3, 5, 10, 13, 14, and 19 indirectly exert IFN-α repression, while BART7-3p and 21-3p indirectly exert IFN-β repression. BART16 downregulates TNF-α expression. Besides, the cascade effects of inflammatory cytokines can be disturbed by EBV-miRNAs, and BHRF1-2-5p and BART6-3p contribute to the immune evasion of cytokine-induced inflammatory response *via* targeting IL-1-receptor 1 and IL-6-receptor, respectively. Symbols: ↓ activation; ⊥ inhibition; ---–>indirect effect.

EBV-miRNAs function as inhibitors of T cell-mediated immunity, thus prompting immune escape [52]. The expression of LMP-1, with strong immunogenicity, can be down-regulated by BART clusters (*i.e*., BART 1-5p, 3, 16, 17-5p, 5-5p, 19-5p, 20) [53], which disturb the immune recognition function of cytotoxic T cells (CTLs). Likewise, the down-regulation of cytokines, such as IL-12, induced by EBV-encoded miRNAs blocks the recognition of EBV-specific CD8^+^ effector T cells [54] (Fig. **3**). In EBV-positive CD4^+^ T cells, BART17 and BHRF1-3 enable EBV to evade the immune surveillance of CD8^+^ T cells by repressing the peptide transporter subunit TAP2 [54]. In EBV-related non-Hodgkin's lymphomas, the upregulation of IFN-inducible T cell-attracting chemokine C-X-C motif chemokine ligand 11(CXCL-11) by BHRF1-3 suppressed the recruitment of cytotoxic T lymphocytes to infection sites during the adaptive immune response [55].

## 4 OTHER STRATEGIES EBV-miRNAs APPLY TO MAINTAIN INFECTION AND REPLICATION

### 4.1 EBV-miRNAs Suppress the Viral Lysis of EBV

The present findings confirm the vital role of EBV-miRNAs in inhibiting viral lytic replication and maintaining latent virus infection. Jung *et al.* proved that BART20-5p from BART cluster 2 could directly down-regulate the expression of BZLF1 and BRLF1 [56]. BART18-5p was demonstrated to suppress the level of MAP3K2, thereby directly targeting BZLF1 and suppressing viral cleavage and replication in the early stage of infection [57].

### 4.2 EBV-miRNAs Modulate Cell Apoptosis

During the early and middle infection periods, EBV encodes specific miRNAs with anti-apoptotic effects. Many recent studies have reported that EBV-encoded miRNAs could interfere with pro-apoptotic genes expression and apoptosis-related gene receptors, such as PUMA, BIM, Caspase3, BAD, TOMM22, and others [24].

Nevertheless, a few EBV-miRNAs (BART5-5p, 15-3p, 16-5p, 17-3p, and 20-3p) inducing apoptosis in EBV-associated gastric carcinoma cells were encoded during the late stages of infection, leading to cell lysis and the release of progeny virus [58, 59].

### 4.3 EBV-miRNAs Promote Tumorigenesis and Metastasis

A growing number of studies have demonstrated the contribution of EBV-miRNAs to pro-tumorigenic inflammation, a vital constituent of the tumor microenvironment (TME) [60]. Secreted by EBV-infected B cells, BART-miRNAs can be released into the peripheral circulation through exosomes and can remotely induce the inflammatory reaction of monocyte and macrophage, leading to increased expression of TNF-α, IL-10, and ARG-1 [61]. BART11 promotes chronic inflammation and carcinogenesis of nasopharyngeal and gastric cancer by inhibiting the anti-tumor effect of forkhead box p1 (FOXP1) [62].

Research has shown a complex relationship between EBV-miRNAs and the production, invasion, and metastasis of EBV-related malignant tumors, which remains to be elucidated. Ma *et al.* showed that suppressed expression of PRDM1, one vital tumor suppressor gene targeted by BHRF1-2, inhibited apoptosis and enhanced the proliferative, invasive, and metastatic capacity in LCL [63]. By contrast, BART6-3p-induced downregulation of LOC553103, a long non-coding RNA, inhibits or even reverses the epithelial-to-mesenchymal transition process, hence repressing the development and metastasis of EBV-positive tumors [59, 64].

## 5 THE SIGNIFICANCE OF EBV-miRNAs AS A POTENTIAL BIOMARKER IN EBV-RELATED FEBRILE DISEASES

### 5.1 Infectious Mononucleosis (IM)

As an acute and self-limited disease, the dynamic expressional profiles of circulating EBV-miRNAs may serve as a breakthrough for disease prediction, diagnosis, and prognosis. Gao *et al*. quantified the EBV-miRNAs in B cells and plasma obtained from children with IM at days 0, 7, and 14. In the very early stages (day 0 and day 7) of primary EBV infection, abundant expression of almost all EBV-miRNAs (especially BART13-1, BHRF1-1, and 1-2-3p), apart from BART2-3P, 20-3p, and 21-5p, indicated active virus replication. Besides, a higher level of BHRF1-derived miRNA expression was found. With the progress of IM, the expressions of almost all EBV-miRNAs, especially BHRF1, were remarkably repressed [65]. These research results are in agreement with others [66-70]. Possible explanations could be the pro-apoptosis role of the early EBV lytic protein BHRF1 in latency III [69, 71].

Hitherto, the distinction between EBV-induced primary IM and EBV-HLH remains a clinical dilemma since they display similar clinical presentations and non-specific indicators [72]. A small-scale experiment analyzing EBV-miRNAs in peripheral blood showed that only BART5-3p and 16 were apparently upregulated during acute infection (after 1-2 months) [70]. The different expression patterns of EBV-miRNAs are considered as biomarkers for differential diagnosis between IM and HLH.

Moreover, the dynamic expression of serum BART16 in EBV-IM displays its potential as a novel biomarker for monitoring IM progression. Additional experiments further suggested that upregulated expression of BART16 directly decreased the level of Cullin Associated And Neddylation Dissociated 1 (CAND1), a known inhibitor of virus replication, and thus dampened innate immunity. The proven mechanism of BART16 indicates that BART16 inhibitors can be therapeutic agents for IM.

### 5.2 Epstein-Barr Virus-associated Hemophagocytic Lymphohistiocytosis (EBV-HLH)

Increasing data support that high expression of inflammatory factors is evidently associated with critical clinical conditions and poor prognosis. It is worth noting that BART3-3p can upregulate IL-6 levels by targeting IPO7, while BART3-3p is highly expressed in EBV-HLH [39]. Thus, BART-derived miRNAs are inferred to induce an over-active inflammatory cytokine response, which consequently leads to a cytokine storm with a persistent high fever. Pro-inflammatory EBV-miRNAs, especially miRNA-BART3-3p, may be potential therapeutic targets for clinical remission and prognosis improvement [73].

As mentioned above, the similarity of clinical manifestation and the absence of specific biomarkers create a clinical conundrum in differential diagnoses between IM and EBV-HLH. One particular study found that the plasma and CD8^+^T cell levels of most BART-encoded miRNAs (BART1-3p, 1-5p, 3-3p, 3-5p, 5-3p, 6-3p, 6-5p, 8-3p, 8-5p, 9-5p, 10-1, 11-3p, 11-5p, 12-1, 13-1, 13*-1, 14-1, 15-1, 16-1, 17-3p, 17-5p, 18-3p, 19-3p, 19-5p, 20-5p, 21-3p and 22) in EBV-triggered HLH were much higher than those in healthy controls and EBV+ IM, suggesting the latent potential of EBV-encoded miRNAs quantification to distinguish EBV-induced HLH from IM [66].

During and after chemotherapy of EBV-HLH, the continuous downward tendency of the plasma BART16-1 levels indicated good prospects of BART16-1 in the evaluation of the EBV-HLH process [66].

### 5.3 Chronic Active EBV Infection (CAEBV)

By comparing the expression of EBV-miRNAs and EBV-DNAs in plasma, Kawano *et al.* suggested BART2-5p, 13, and 15 as possible molecular biomarkers of the prognosis and severity assessment of CAEBV [70]. In contrast to the failure of plasma EBV-DNA loads in differentiating between active and inactive thresholds, the detection of plasma BART13 levels can clearly determine whether CAEBV is in the active phase. BART2-5p and 15 can identify whether CAEBV achieves complete remission. The experiment also showed particularly higher levels of serum BART1-5p, 2-5p, 5, and 22 in CAEBV-T/NK in contrast to those in IM and the healthy control group. Therefore, serum BART1-5p, 2-5p, 5, and 22 were supposed to be useful adjuncts to discriminate between two diseases with similar clinical presentations. Moreover, plasma levels of BART2-5p, 4, 7, 13, 15, and 22 in CAEBV-T/NK patients suffering the active, progressing state were significantly higher than those in an inactive state [70].

A comprehensive viral miRNA detection in children with CAEBV, EBV-HLH and EBV+ NPC suggested the abundant vial miRNA expression of BART1-5p, 3-3p, 4-5p, 6-3p, 7-3p, 13-3p, 15, 16, and 19-3p. Furthermore, BART4-5p and 19-3p were suggested to be potential targets for EBV-associated diseases treatment due to their contribution to tumorigenesis. In children with CAEBV and HLH, the abundant BART19-3p directly downregulated the mRNA level of adenomatous polyposis coli (APC), a Wnt/β-catenin signaling regulatory gene. BART4-5p was speculated to promote tumorigenesis by inhibiting BH3-interacting domain death agonist (BID) [74].

### 5.4 EBV-associated Tumors

Both Hodgkins and non-Hodgkins lymphoma induced by EBV, including diffuse large B-cell lymphoma (DLBCL), extranodal T/NK cell lymphoma, nasal type (ENKTL), peripheral T-cell lymphoma, unspecified (PTCL-U), and post-transplant lymphoproliferative disorder (PTLD), can induce typical B symptoms (prolonged fever, unintentional weight loss, and drenching night sweats). A growing amount of literature supports the potential of specific-expressed EBV-miRNAs to distinguish these malignant diseases from benign lymphoproliferative disorders.

A strong positive correlation was proven between BART-miRNAs and EBV-positive B lymphoma in L591 cells obtained from biopsy tissues with EBV-positive DLBCL. The specific overexpression of miRNA-BART13 indicated its diagnostic and therapeutic potential [61]. Over-expressions of BART7, 22, 10, 11-5p and 16 were detected in tissue samples of EBV-positive DLBCL, suggesting its potential for discrimination [75]. In EBV-positive DLBCL, BHRF1-2-5p was observed to down-regulate slightly the expression of PD-L1/L2 surface proteins, thus weakening the induction of PD-L1/L2 by LMP-1. Consequently, BHRF1-2-5p is considered a potential diagnostic and therapeutic target in DLBCL [45]. CXCL11 is an IFN-induced chemokine receptor on T cells, permitting resistance to the cytotoxic effect of T/NK cells. BART2 and BHRF1-3 could inhibit CXCL11 to dampen host immunity in AIDS-associated DLBCL. The singular over-expression of BHRF1-1 may be helpful in the clinical diagnosis of DLBCL complicated with pyothorax [55].

According to 2016 WHO classification, malignant T/NK cell diseases caused by EBV include ENKTL, primary EBV positive lymph node T/NK cell lymphoma (tentative), including PTCL-U, and others. Komabayashi *et al.* confirmed the remarkably increased level of BART1-5p, 2-5p, 7-3p and 13-3p in EBV-associated cell lines derived from ENKTL, SNK6, and SNT16. BART1-5p, 2-5p, 7-3p and 13-3p were closely related to the development and poor prognosis of ENKTL and could accurately distinguish ENKTL patients from healthy controls [76]. Interestingly, Alles *et al*. observed the highest expression levels of miRNA-BART8, 10, 19 and 22 [52], while Ramakrishnan R *et al.* observed the highest expression levels of miRNA-BART1, 7, 16 and 17 [77]. Such differences might be due to the application of diverse sequencing methods (sequencing vs. microarray). T-bet is a T-box transcription factor Th1 cells utilize to trigger immune cell differentiation, thus mediating the secretion of cytokines, such as TNF-α, IFN-γ, IL-2, and IL-10 [78]. BART20-5p was considered a potential biomarker for ENKTL treatment due to its immune inhibition *via* decreasing T-bet expression [79].

Previous clinical analyses revealed ENKTL as one of the secondary complications to HLH and CAEBV. Abundant expression of type-I IFN signaling inhibitors, BART1-5p, 2-5p, 7, 13-3p, 16 and 22, was found in the peripheral blood sample with CAEBV and ENKTL, indicating their potential to be warning signs for poor prognosis [70, 76].

Further, EBV + PTCL has been shown to be in latent phase II. In EBV+ PTCL samples, the expression of all detected BARTs (*i.e*., BART1-5p, 2, 7-5p, and 10-5p) was observed [80].

In addition, a strong association between EBV infection and PTLD has been demonstrated [81]. In almost all EBV-related PTLD, BARTs constituted the majority of the high-expression group [82]. It was proposed that expression levels of plasma BART2-5p and BHRF1-2-5p could be utilized as potential biomarkers in the detection of PTLD risk in pediatric renal transplant recipients [68].

## CONCLUSION

Even though there are many advanced molecular biological methods available for diagnosis, it is still an issue for physicians to distinguish different types of EBV infection in a timely and accurate manner, which is related to the prognosis of critical diseases [83]. Conventional methods for EBV detection mainly include *in situ* hybridization (ISH), immunohistochemistry (IHC), and serological testing, like anti-EBV nuclear antigen-1(EBNA1) immunoglobulin G detection [84-86]. Nevertheless, many of the current diagnostic methods for EBV detection have limitations [31]. For example, in terms of serological methods, the sensitivity of heterophilic antibody test is relatively low, while viral capsid antigens (VCA) and EBNA antibody tests are more expensive and require much more time [33].

Thus far, there have been no satisfactory therapies for EBV-related diseases with fever. Antiviral drugs (including acyclovir, valaciclovir, or ganciclovir) and glucocorticoid for treating IM have raised concerns due to limited efficacy and potential adverse effects [29, 30]. Allogeneic hematopoietic stem cell transplantation (allo-HSCT), the radical cure for HLH and CAEBV, meets obstruction due to its high expense and risk of post-transplant complications [87-89].

The specific viral miRNA expression profiles in EBV-related fevers suggest the potential value of EBV-miRNAs as molecular biomarkers to assist in the identification, diagnosis and prognosis of EBV-related fever, as well as therapeutic targets for drug development (Table **1**) [90]. With regard to IM, scholars currently focus on the detection of early infections. The combination of EBV-miRNA detection and clinical signs might be more helpful in judging the infectious process of patients, especially in the early phases. Also, in the context of CAEBV, a notable finding was that some EBV-encoded miRNAs play a part in guiding disease prognoses. Compared to only detecting EBV loads, evaluation of both viral miRNA and DNA can be more helpful in the context of managing clinical medication and treatment. The detection of some specific EBV-miRNAs may assist in preliminarily diagnosis and treatment direction.

A growing body of literature on exosomes has proved the essential role of plasma EBV-miRNAs in intercellular signal transduction [91]. Plasma exosomes from EBV-infected lymphoma cells, which mainly contain BART-miRNAs, function similarly to pro-inflammatory cytokine to trigger the secretion of ARG1, TNF-α, and IL-10 in monocytes and macrophages. In this way, inflammatory responses of tumor-associated macrophages could be activated, supporting the growth, proliferation, migration, and immune response of lymphoma [28, 92, 93]. Targeted or off-targeted delivery of miRNAs as well as miRNA inhibitors may be a major trend in future therapeutic development [61, 86]. Further research should be carried out to detect new inflammatory targets of EBV-miRNAs and to reveal the regulatory network between EBV-encoded miRNAs and target genes. The screening and identification of viral miRNAs with potential therapeutic effects for EBV-related fever may conduce to the development of novel therapeutic approaches for such diseases.

New vaccines are under development to reduce the incidence of infectious mononucleosis [94]. As microRNAs have been studied as potential biomarkers for candidate vaccines for different types of viral infections, such as respiratory syncytial virus [95], the latent potential of EBV microRNAs to improve vaccines for EBV-related diseases remains to be explored.

Prior to clinical application, biomarkers require considerable research to validate their specificity, stability, detectability, and accessibility [16]. As short RNA molecules of ∼22nt in length, circulating miRNAs are expressed stably both *in vivo* and *in vitro* [96]. Previous experiments have also shown that numerous viral miRNAs can be obtained from peripheral blood circulation by such non-invasive means [13]. EBV-miRNAs are still at their early stages as ideal diagnostic, prognostic, or monitoring biomarkers in detecting febrile patients. (1) Given the low complementary ratio and manifold targets of miRNAs [97], the complex regulatory network mechanism formed between EBV-miRNAs and the host is still unclear. Further participation of proteomics, genomics, and transcriptomics may explain the thermogenic mechanism of EBV-miRNA. (2) Most literature on EBV-miRNA in diseases only concentrates on the special expression of certain miRNAs. Future improvement in the diagnostic specificity of miRNAs calls for large-scale field trials with better reproducibility, minimal sampling factors (*e.g*., age, sex, past medical history), as well as diversity in both technical means (*e.g*., sample preservation, pre-processing before sequencing, detection methods) and statistic methods. Whether EBV-miRNAs exhibit greater diagnostic significance before the development of diseases or at the onset of diseases has to be explored. Additionally, exploration of whether EBV-miRNAs can be used as sensitive indicators of EBV-infection-induced complications also warrants further study. (3) The sequence similarity analysis revealed that EBV-miRNAs shared high similarities with human miRNAs, which may interfere with the sequencing results [98]. Advances in QT-PCR technology and other new detection methods (*i.e*., rolling circle amplification, surface-enhanced Raman scattering, Agilent 2100 Bioanalyzer) may provide more accurate results [99, 100]. (4) Different expression profile of the same disease in various population samples and laboratories shows the necessity of unifying EBV detection methods. (5) Till now, the development of 5 miRNA drugs has been discontinued or suspended due to treatment-related side effects [101-106]. The safety and efficacy of EBV-miRNAs as therapeutic targets also need verification in the future.

## Figures and Tables

**Table 1:** Summary of the EBV-miRNAs as potential biomarkers in EBV-related diseases and the indication of their aberrant expression*.

**EBV-miRNA**	**Abnormal Expression**	**Related Diseases**	**Indication**
BART5-3p,16	Over-expression	IM	Early period of infection
BART2-5p,13,15	Increased or decreased level of expression	CAEBV	Indicating prognosis
BART3-3p	Over-expression	EBV-HLH	Indicating EBV-HLH, distinguishing from IM
BART19-3p	Over-expression	EBV-HLH, CAEBV, NPC	A potential therapeutic target
BART7,22,10, 11-5p	High detection rate	DLBCL	Indicating DLBCL
BHRF1-1	Over-expression	PAL	Indicating PAL
BART1-5p, 2-5p. 7-3p, 13-3	Increased level of expression	ENKTL	Indicating development and poor prognosis
BART1-5p, 2, 5, 7-5p, 10-5p	Appearance	PTCL-U	
BART7-3p	Over-expression	Malignant tumor	Indicating malignant tumor
BART1-3p,3-3p,4-5p,5-5p,9-3p	Over-expression	Epithelial cell tumor	Indicating epithelial cell tumor
BART6-3p,8-3p	Over-expression	NPC	Indicating NPC
BART2-5p, BHRF1-2-5p	High detection rate	PTLD	Potential biomarkers to detect the risk of PTDL in pediatric renal transplant recipients

**Note:** *[21, 39, 68, 70, 75, 76, 80, 90]. **Abbreviations:** IM, infectious mononucleosis; CAEBV, chronic active EBV infection; EBV-HLH, Epstein-Barr virus-associated hemophagocytic lymphohistiocytosis; PAL, pyothorax-associated lymphoma; ENKTL, extranodal T/NK cell lymphoma, nasal type; PTCL-U, peripheral T-cell lymphoma, unspecified; DLBCL, diffuse large B-cell lymphoma; NPC, nasopharyngeal carcinoma; PTLD, post-transplant lymphoproliferative disorder.

## LIST OF ABBREVIATIONS

EBV: Epstein - Barr virus
IM: Infectious mononucleosis
EBV-HLH: Epstein-Barr Virus-associated hemophagocytic lymphohistiocytosis

## References

[1] Lieberman P.M. (2014). Virology. Epstein-Barr virus turns 50.. Science.

[2] Münz C. (2019). Latency and lytic replication in Epstein–Barr virus-associated oncogenesis.. Nat. Rev. Microbiol..

[3] Young L.S., Rickinson A.B. (2004). Epstein–Barr virus: 40 years on.. Nat. Rev. Cancer.

[4] Schmidt C.W., Misko I.S. (1995). The ecology and pathology of Epstein-Barr virus.. Immunol. Cell Biol..

[5] Kerr J.R. (2019). Epstein-Barr virus (EBV) reactivation and therapeutic inhibitors.. J. Clin. Pathol..

[6] McKenzie J., El-Guindy A. (2015). Epstein-barr virus lytic cycle reactivation.. Curr. Top. Microbiol. Immunol..

[7] Hammerschmidt W., Sugden B. (1988). Identification and characterization of oriLyt, a lytic origin of DNA replication of Epstein-Barr virus.. Cell.

[8] Schepers A., Pich D., Hammerschmidt W. (1996). Activation of oriLyt, the lytic origin of DNA replication of Epstein-Barr virus, by BZLF1.. Virology.

[9] Pfeffer S., Zavolan M., Grässer F.A. (2004). Identification of virus-encoded microRNAs.. Science.

[10] Murer A., Rühl J., Zbinden A. (2019). MicroRNAs of epstein-barr virus attenuate T-cell-mediated immune control in vivo.. MBio.

[11] Lu T.X., Rothenberg M.E., Micro R.N.A. (2018). MicroRNA.. J. Allergy Clin. Immunol..

[12] Lünemann A., Rowe M., Nadal D. (2015). Innate immune recognition of EBV.. Curr. Top. Microbiol. Immunol..

[13] Saliminejad K., Khorram Khorshid H.R., Soleymani Fard S., Ghaffari S.H. (2019). An overview of microRNAs: Biology, functions, therapeutics, and analysis methods.. J. Cell. Physiol..

[14] Dong H., Lei J., Ding L. (2013). MicroRNA: Function, detection, and bioanalysis.. Chem. Rev..

[15] Vargas A.J., Harris C.C. (2016). Biomarker development in the precision medicine era: Lung cancer as a case study.. Nat. Rev. Cancer.

[16] Biomarker Working F.D.A-N.I.H.G. (2016). BEST (Biomarkers, EndpointS, and other Tools)..

[17] Biomarkers Definitions Working Group (2001). Biomarkers and surrogate endpoints: Preferred definitions and conceptual framework.. Clin. Pharmacol. Ther..

[18] Barth S., Meister G., Grässer F.A. (2011). EBV-encoded miRNAs.. Biochim. Biophys. Acta. Gene Regul. Mech..

[19] Klinke O., Feederle R., Delecluse H.J. (2014). Genetics of Epstein-Barr virus microRNAs.. Semin. Cancer Biol..

[20] Navari M., Fuligni F., Laginestra M.A. (2014). Molecular signature of Epstein Barr virus-positive Burkitt lymphoma and posttransplant lymphoproliferative disorder suggest different roles for Epstein Barr virus.. Front. Microbiol..

[21] Sakamoto K., Sekizuka T., Uehara T. (2017). Next-generation sequencing of miRNAs in clinical samples of Epstein-Barr virus-associated B-cell lymphomas.. Cancer Med..

[22] Amoroso R., Fitzsimmons L., Thomas W.A., Kelly G.L., Rowe M., Bell A.I. (2011). Quantitative studies of Epstein-Barr virus-encoded microRNAs provide novel insights into their regulation.. J. Virol..

[23] Kanda T. (2018). EBV-encoded latent genes.. Adv. Exp. Med. Biol..

[24] Wang M., Gu B., Chen X., Wang Y., Li P., Wang K. (2019). The function and therapeutic potential of epstein-barr virus-encoded MicroRNAs in cancer.. Mol. Ther. Nucleic Acids.

[25] Wang M., Yu F., Wu W., Wang Y., Ding H., Qian L. (2018). Epstein-Barr virus-encoded microRNAs as regulators in host immune responses.. Int. J. Biol. Sci..

[26] Kalluri R., LeBleu V.S. (2020). The biology, function, and biomedical applications of exosomes.. Science.

[27] Chen W., Xie Y., Wang T., Wang L. (2021). New insights into Epstein Barr virus associated tumors: Exosomes (Review).. Oncol. Rep..

[28] Pegtel D.M., Cosmopoulos K., Thorley-Lawson D.A. (2010). Functional delivery of viral miRNAs via exosomes.. Proc. Natl. Acad. Sci. USA.

[29] De Paor M., O’Brien K., Fahey T., Smith S.M. (2016). Antiviral agents for infectious mononucleosis (glandular fever).. Cochrane Libr..

[30] Luzuriaga K., Sullivan J.L. (2010). Infectious mononucleosis.. N. Engl. J. Med..

[31] Abusalah M.A.H., Gan S.H., Al-Hatamleh M.A.I., Irekeola A.A., Shueb R.H., Yean Yean C. (2020). Recent advances in diagnostic approaches for epstein–barr virus.. Pathogens.

[32] Kimura H., Kwong Y.L. (2019). EBV viral loads in diagnosis, monitoring, and response assessment.. Front. Oncol..

[33] Fugl A., Andersen C.L. (2019). Epstein-Barr virus and its association with disease - a review of relevance to general practice.. BMC Fam. Pract..

[34] Aggarwal B.B., Shishodia S., Sandur S.K., Pandey M.K., Sethi G. (2006). Inflammation and cancer: How hot is the link?. Biochem. Pharmacol..

[35] Fajgenbaum D.C., June C.H. (2020). Cytokine storm.. N. Engl. J. Med..

[36] Kasahara Y., Yachie A., Takei K. (2001). Differential cellular targets of Epstein-Barr virus (EBV) infection between acute EBV-associated hemophagocytic lymphohistiocytosis and chronic active EBV infection.. Blood.

[37] Bay A., Coskun E., Oztuzcu S., Ergun S., Yilmaz F., Aktekin E. (2013). Evaluation of the plasma micro RNA expression levels in secondary hemophagocytic lymphohistiocytosis.. Mediterr. J. Hematol. Infect. Dis..

[38] Ungerleider N., Bullard W., Kara M. (2021). EBV miRNAs are potent effectors of tumor cell transcriptome remodeling in promoting immune escape.. PLoS Pathog..

[39] Dölken L., Malterer G., Erhard F. (2010). Systematic analysis of viral and cellular microRNA targets in cells latently infected with human gamma-herpesviruses by RISC immunoprecipitation assay.. Cell Host Microbe.

[40] Yang I.V., Wade C.M., Kang H.M. (2009). Identification of novel genes that mediate innate immunity using inbred mice.. Genetics.

[41] Haneklaus M., Gerlic M., Kurowska-Stolarska M. (2012). Cutting Edge: MiR-223 and EBV miR-BART15 Regulate the NLRP3 Inflammasome and IL-1β Production.. J. Immunol..

[42] Ambrosio M.R., Navari M., Di Lisio L. (2014). The epstein barr-encoded BART-6-3p microRNA affects regulation of cell growth and immuno response in Burkitt lymphoma.. Infect. Agent. Cancer.

[43] Skinner C.M., Ivanov N.S., Barr S.A., Chen Y., Skalsky R.L. (2017). An epstein-barr virus MicroRNA blocks interleukin-1 (IL-1) signaling by targeting IL-1 receptor 1.. J. Virol..

[44] Hooykaas M.J.G., van Gent M., Soppe J.A. (2017). EBV MicroRNA BART16 suppresses type I IFN signaling.. J. Immunol..

[45] Cristino A.S., Nourse J., West R.A. (2019). EBV microRNA-BHRF1-2-5p targets the 3′UTR of immune checkpoint ligands PD-L1 and PD-L2.. Blood.

[46] Lu Y., Qin Z., Wang J. (2017). Epstein-barr virus miR-BART6-3p inhibits the RIG-I pathway.. J. Innate Immun..

[47] Ghasemi F., Gameiro S.F., Tessier T.M., Maciver A.H., Mymryk J.S. (2020). High levels of class I major histocompatibility complex mRNA are present in epstein–barr virus-associated gastric adenocarcinomas.. Cells.

[48] Huang W.T., Lin C.W. (2014). EBV-encoded miR-BART20-5p and miR-BART8 inhibit the IFN-γ-STAT1 pathway associated with disease progression in nasal NK-cell lymphoma.. Am. J. Pathol..

[49] Skalsky R.L., Corcoran D.L., Gottwein E. (2012). The viral and cellular microRNA targetome in lymphoblastoid cell lines.. PLoS Pathog..

[50] Harding C.V. (1991). Pathways of antigen processing.. Curr. Opin. Immunol..

[51] Tagawa T., Albanese M., Bouvet M. (2016). Epstein-Barr viral miRNAs inhibit antiviral CD4+ T cell responses targeting IL-12 and peptide processing.. J. Exp. Med..

[52] Alles J., Menegatti J., Motsch N. (2016). miRNA expression profiling of Epstein-Barr virus-associated NKTL cell lines by Illumina deep sequencing.. FEBS Open Bio.

[53] Verhoeven R.J.A., Tong S., Zhang G. (2016). NF-κB signaling regulates expression of epstein-barr virus bart micrornas and long noncoding rnas in nasopharyngeal carcinoma.. J. Virol..

[54] Albanese M., Tagawa T., Bouvet M. (2016). Epstein–Barr virus microRNAs reduce immune surveillance by virus-specific CD8+ T cells.. Proc. Natl. Acad. Sci..

[55] Xia T., O’Hara A., Araujo I. (2008). EBV microRNAs in primary lymphomas and targeting of CXCL-11 by ebv-mir-BHRF1-3.. Cancer Res..

[56] Jung Y.J., Choi H., Kim H., Lee S.K. (2014). MicroRNA miR-BART20-5p stabilizes Epstein-Barr virus latency by directly targeting BZLF1 and BRLF1.. J. Virol..

[57] Qiu J., Cosmopoulos K., Pegtel M. (2011). A novel persistence associated EBV miRNA expression profile is disrupted in neoplasia.. PLoS Pathog..

[58] Choi H., Lee S.K. (2017). TAX1BP1 downregulation by EBV-miR-BART15-3p enhances chemosensitivity of gastric cancer cells to 5-FU.. Arch. Virol..

[59] Choi H., Lee H., Kim S.R., Gho Y.S., Lee S.K. (2013). Epstein-Barr virus-encoded microRNA BART15-3p promotes cell apoptosis partially by targeting BRUCE.. J. Virol..

[60] Zuo L., Yue W., Du S. (2017). An update: Epstein-Barr virus and immune evasion via microRNA regulation.. Virol. Sin..

[61] Higuchi H., Yamakawa N., Imadome K.I. (2018). Role of exosomes as a proinflammatory mediator in the development of EBV-associated lymphoma.. Blood.

[62] Song Y., Li X., Zeng Z. (2016). Epstein-Barr virus encoded miR-BART11 promotes inflammation-induced carcinogenesis by targeting FOXP1.. Oncotarget.

[63] Ma J., Nie K., Redmond D. (2016). EBV-miR-BHRF1-2 targets PRDM1/Blimp1: Potential role in EBV lymphomagenesis.. Leukemia.

[64] He B., Li W., Wu Y. (2016). Epstein-Barr virus-encoded miR-BART6-3p inhibits cancer cell metastasis and invasion by targeting long non-coding RNA LOC553103.. Cell Death Dis..

[65] Gao L., Ai J., Xie Z. (2015). Dynamic expression of viral and cellular microRNAs in infectious mononucleosis caused by primary Epstein-Barr virus infection in children.. Virol. J..

[66] Zhou C., Xie Z., Gao L. (2015). Profiling of EBV-encoded microRNAs in EBV-associated hemophagocytic lymphohistiocytosis.. Tohoku J. Exp. Med..

[67] Hartung A., Makarewicz O., Egerer R. (2019). EBV miRNA expression profiles in different infection stages: A prospective cohort study.. PLoS One.

[68] Hassan J., Dean J., De Gascun C.F. (2018). Plasma EBV microRNAs in paediatric renal transplant recipients.. J. Nephrol..

[69] Pearson G.R., Luka J., Petti L. (1987). Identification of an Epstein-Barr virus early gene encoding a second component of the restricted early antigen complex.. Virology.

[70] Kawano Y., Iwata S., Kawada J. (2013). Plasma viral microRNA profiles reveal potential biomarkers for chronic active Epstein-Barr virus infection.. J. Infect. Dis..

[71] Seto E., Moosmann A., Grömminger S., Walz N., Grundhoff A., Hammerschmidt W. (2010). Micro RNAs of Epstein-Barr virus promote cell cycle progression and prevent apoptosis of primary human B cells.. PLoS Pathog..

[72] Shi J., Chu C., Yu M. (2021). Clinical warning of hemophagocytic syndrome caused by Epstein-Barr virus.. Ital. J. Pediatr..

[73] Han X., Ye Q., Zhang W., Tang Y., Xu X., Zhang T. (2017). Cytokine profiles as novel diagnostic markers of Epstein-Barr virus–associated hemophagocytic lymphohistiocytosis in children.. J. Crit. Care.

[74] Zhang Q., Luo D., Xie Z., He H., Duan Z. (2020). The oncogenic role of miR-BART19-3p in epstein-barr virus-associated diseases.. BioMed Res. Int..

[75] Imig J., Motsch N., Zhu J.Y. (2011). microRNA profiling in Epstein–Barr virus-associated B-cell lymphoma.. Nucleic Acids Res..

[76] Komabayashi Y., Kishibe K., Nagato T., Ueda S., Takahara M., Harabuchi Y. (2017). Circulating Epstein-Barr virus-encoded micro-RNAs as potential biomarkers for nasal natural killer/T-cell lymphoma.. Hematol. Oncol..

[77] Ramakrishnan R., Donahue H., Garcia D. (2011). Epstein-Barr virus BART9 miRNA modulates LMP1 levels and affects growth rate of nasal NK T cell lymphomas.. PLoS One.

[78] Levine A.G., Mendoza A., Hemmers S. (2017). Stability and function of regulatory T cells expressing the transcription factor T-bet.. Nature.

[79] Lin T.C., Liu T.Y., Hsu S.M., Lin C.W. (2013). Epstein-Barr virus-encoded miR-BART20-5p inhibits T-bet translation with secondary suppression of p53 in invasive nasal NK/T-cell lymphoma.. Am. J. Pathol..

[80] Jun S.M., Hong Y.S., Seo J.S., Ko Y.H., Yang C.W., Lee S.K. (2008). Viral microRNA profile in Epstein-Barr virus-associated peripheral T cell lymphoma.. Br. J. Haematol..

[81] Marques-Piubelli M.L., Salas Y.I., Pachas C., Becker-Hecker R., Vega F., Miranda R.N. (2020). Epstein–Barr virus-associated B-cell lymphoproliferative disorders and lymphomas: A review.. Pathology.

[82] Fink S.E.K., Gandhi M.K., Nourse J.P. (2014). A comprehensive analysis of the cellular and EBV-specific microRNAome in primary CNS PTLD identifies different patterns among EBV-associated tumors.. Am. J. Transplant..

[83] Unger M., Karanikas G., Kerschbaumer A., Winkler S., Aletaha D. (2016). Fever of unknown origin (FUO) revised.. Wien. Klin. Wochenschr..

[84] Baer R., Bankier A.T., Biggin M.D. (1984). DNA sequence and expression of the B95-8 Epstein—Barr virus genome.. Nature.

[85] Mundo L., Ambrosio M.R., Picciolini M. (2017). Unveiling another missing piece in EBV-driven lymphomagenesis: EBV-encoded MicroRNAs expression in EBER-negative burkitt lymphoma cases.. Front. Microbiol..

[86] Navari M., Etebari M., Ibrahimi M., Leoncini L., Piccaluga P. (2018). Pathobiologic roles of epstein–barr virus-encoded MicroRNAs in human lymphomas.. Int. J. Mol. Sci..

[87] Ohga S., Kudo K., Ishii E. (2010). Hematopoietic stem cell transplantation for familial hemophagocytic lymphohistiocytosis and Epstein-Barr virus-associated hemophagocytic lymphohistiocytosis in Japan.. Pediatr. Blood Cancer.

[88] Cohen J.I., Jaffe E.S., Dale J.K. (2011). Characterization and treatment of chronic active Epstein-Barr virus disease: A 28-year experience in the United States.. Blood.

[89] Fujiwara S., Kimura H., Imadome K. (2014). Current research on chronic active Epstein-Barr virus infection in Japan.. Pediatr. Int..

[90] Kaul V., Weinberg K.I., Boyd S.D. (2018). Dynamics of viral and host immune cell MicroRNA expression during acute infectious mononucleosis.. Front. Microbiol..

[91] Koppers-Lalic D., Hogenboom M.M., Middeldorp J.M., Pegtel D.M. (2013). Virus-modified exosomes for targeted RNA delivery; A new approach in nanomedicine.. Adv. Drug Deliv. Rev..

[92] Kotani A. (2014). The role of tumor-derived secretary small RNAs in EBV related lymphoma.. Uirusu.

[93] Meckes D.G., Shair K.H.Y., Marquitz A.R., Kung C.P., Edwards R.H., Raab-Traub N. (2010). Human tumor virus utilizes exosomes for intercellular communication.. Proc. Natl. Acad. Sci..

[94] Cohen J.I. (2018). Vaccine development for epstein-barr virus.. Adv. Exp. Med. Biol..

[95] Atherton L.J., Jorquera P.A., Bakre A.A., Tripp R.A. (2019). Determining immune and miRNA biomarkers related to Respiratory Syncytial Virus (RSV) vaccine types.. Front. Immunol..

[96] Turchinovich A., Weiz L., Langheinz A., Burwinkel B. (2011). Characterization of extracellular circulating microRNA.. Nucleic Acids Res..

[97] Wang J., Chen J., Sen S. (2016). MicroRNA as biomarkers and diagnostics.. J. Cell. Physiol..

[98] Chen S.J., Chen G.H., Chen Y.H. (2010). Characterization of Epstein-Barr virus miRNAome in nasopharyngeal carcinoma by deep sequencing.. PLoS One.

[99] Kobori T., Takahashi H. (2014). Expanding possibilities of rolling circle amplification as a biosensing platform.. Anal. Sci..

[100] Alhasan A.H., Kim D.Y., Daniel W.L. (2012). Scanometric microRNA array profiling of prostate cancer markers using spherical nucleic acid-gold nanoparticle conjugates.. Anal. Chem..

[101] Zhang S., Cheng Z., Wang Y., Han T. (2021). The risks of miRNA therapeutics: In a drug target perspective.. Drug Des. Devel. Ther..

[102] Balfour H.H., Dunmire S.K., Hogquist K.A. (2015). Infectious mononucleosis.. Clin. Transl. Immunology.

[103] Young L.S., Yap L.F., Murray P.G. (2016). Epstein–Barr virus: More than 50 years old and still providing surprises.. Nat. Rev. Cancer.

[104] Dunmire S.K., Verghese P.S., Balfour H.H. (2018). Primary Epstein-Barr virus infection.. J. Clin. Virol..

[105] Kempkes B., Robertson E.S. (2015). Epstein-Barr virus latency: Current and future perspectives.. Curr. Opin. Virol..

[106] Hammerschmidt W. (2015). The epigenetic life cycle of epstein–barr virus.. Curr. Top. Microbiol. Immunol..

